# Supplementary figures and images for: Longitudinal deep sequencing informs vector selection and future deployment strategies for transmissible vaccines
Source: PLoS Biol. 2022 Apr 19;20(4):e3001580. doi: 10.1371/journal.pbio.3001580 (PMC9017877; doi:10.1371/journal.pbio.3001580)

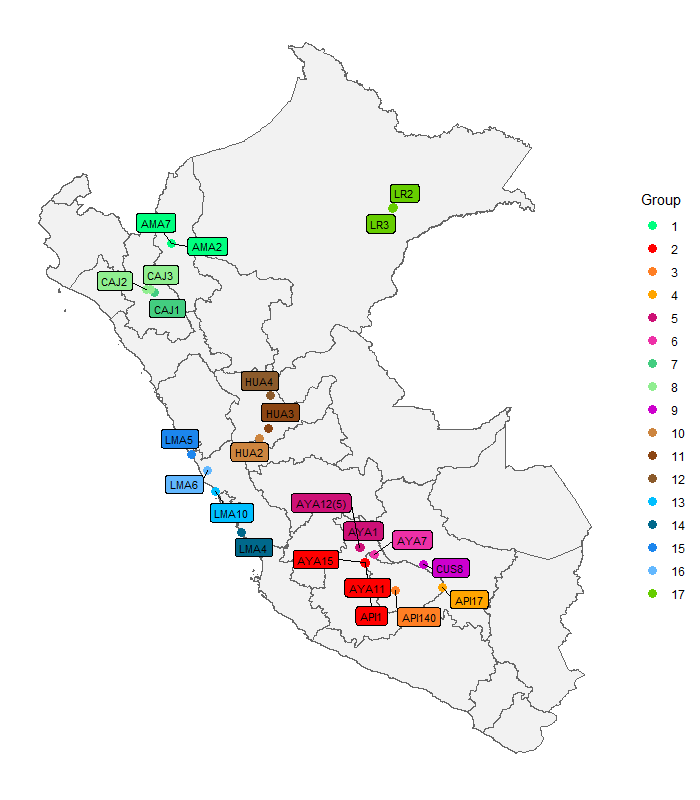

Supplement: S1 Fig — Map of Peru showing the grouping of sample colonies within 10 km of each other into 17 groups (base map: https://gadm.org/maps/PER.html). (TIFF) [file pbio.3001580.s001.tiff]

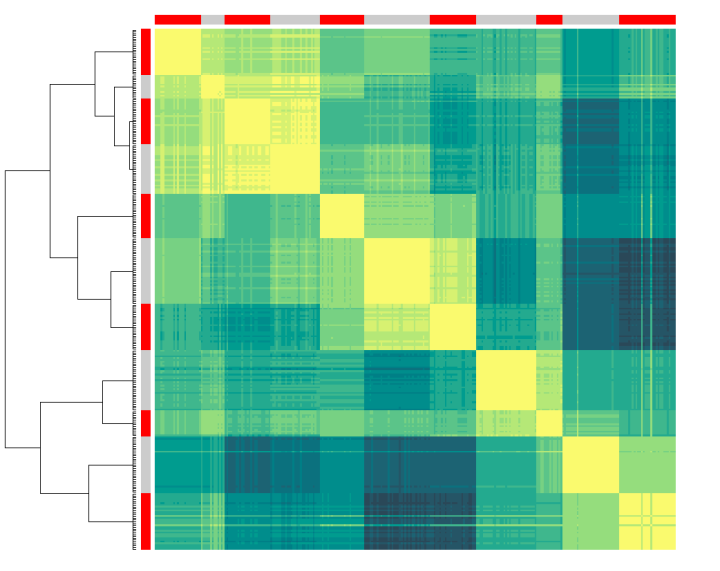

Supplement: S2 Fig — Plot of the 11 clusters produced by affinity propagation clustering and the similarity of each sequence (yellow = high similarity, dark blue = low similarity). Grey and red bars are used to separate clusters. The dendrogram shows relatedness between the clusters. Data underlying this figure can be found in S6 Data. DrBHV, Desmodus rotundus betaherpesvirus. (TIFF) [file pbio.3001580.s002.tiff]

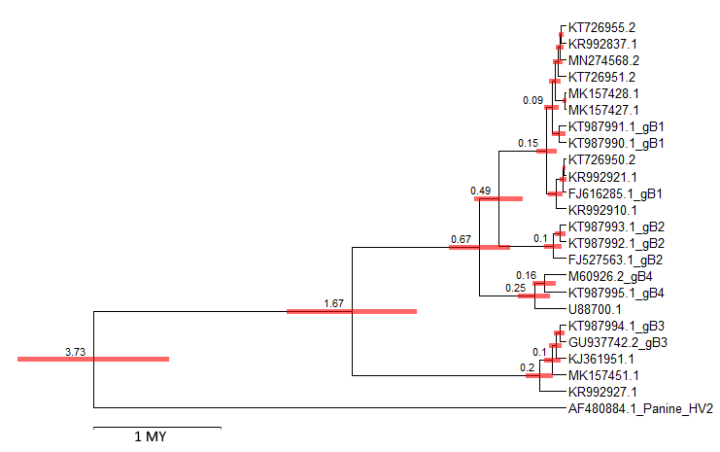

Supplement: S3 Fig — Bayesian phylogeny of HCMV glycoprotein B sequences with the age of nodes (million years). Panine herpesvirus 2 used as an outgroup with a set divergence date of 3.8 mya based on the divergence date of host species. Node bars represent 95% highest posterior densities for date estimates. Data underlying this figure can be found in S7 Data. HCMV, human cytomegalovirus. (TIFF) [file pbio.3001580.s003.tiff]
