# Supplementary material for: Longitudinal deep sequencing informs vector selection and future deployment strategies for transmissible vaccines
Source: PLoS Biol. 2022 Apr 19;20(4):e3001580. doi: 10.1371/journal.pbio.3001580 (PMC9017877; doi:10.1371/journal.pbio.3001580)
Supplement: S1 Text — (DOCX) [file pbio.3001580.s009.docx]

**Supporting text**

**Comparison of host and virus population structures**

Microsatellite sequencing of *D. rotundus* samples was carried out previously (1,2), and the data re-analysed for this study using GenAlEx (3) to calculate pairwise F_ST_ values. Data were divided by group, with only those from groups also tested for DrBHV retained in the final dataset. Multi-dimensional scaling plots of the F_ST_ values were visualised in R.

The Least Cost Distance (LCD) model of colonies in Peru based on distance and elevation data were calculated using the *‘geosphere’* (4) and *‘gdistance’* (5) and *‘raster’* (6) packages in R. Three models were run and tested for correlation with DrBHV F_ST_; (i) a simple model of same cost below the cut-off (3600m; the maximum observed elevation for vampire bat roosts (2)), with movement above this height impossible, (ii) a linear increase of cost of movement with elevation until the cut-off, and (iii) an exponential increase of cost with elevation. Model (ii) showed the highest Mantel correlation with DrBHV FST, and the resulting distance matrix was used to produce the dendrogram (Fig 3C) using *‘ggtree’* (7). This was closely followed by model (i), and model (iii) had a much lower Mantel correlation.

Mantel tests in the ‘*vegan*’ package of R (8) were used to evaluate correlations among pairwise distance matrices calculated from DrBHV Fst values, bat microsatellites and least cost distance matrices of landscape isolation. Where necessary matrices were simplified to account for missing data from some colonies.

**qPCR of DrBHV longitudinally collected samples**

Primers and probe were designed using IDT primer design. DrBHV primers: BHV-8F: 5’-TTCATCTCGTCCACCAACAC-3’, BHV-8R: 5’-CGATGGTCTCGTCCATGAAG-3’; DrBHV probe: 5’-6-FAM- ACAAGCCCACCTTCATCACCATCA-BHQ1-3’. The probe was HPLC purified.

The primers were used at a final concentration of 100nM, and the probe at 200nM. Master mixes were made using Agilent Brilliant III ultra-fast qPCR master mix as per the manufacturer’s instructions, including a 1:500 dilution of the reference dye. The qPCR protocol was carried out on the ABI7500 Fast machine, with 3 replicates of each reaction. The protocol was as follows: 95°C for 3 minutes followed by 40 cycles of 95°C for 12s and 60°C for 30s. A PCR-product positive control of known concentration for DrBHV was used in each run to normalise the baseline and threshold Ct values for comparisons between runs. The positive control was made and added to plates in a separate room to minimise cross-contamination.

Pearson’s correlation showed a negative relationship between sample Ct values and the number of haplotypes detected by sequencing (R = -0.39, p-value = 0.013). This shows that higher viral loads correspond to a greater number of haplotypes, suggesting a relationship between intra-host viral diversity and the amount of virus shedding. Full Ct values are linked under data availability.

**Age determination of glycoprotein B genotypes in DrBHV and HCMV**

The bam alignments from bat samples that had a single strain infection of DrBHV were used to form consensus sequences spanning the entire 12kb region, or as much as possible given the available sequencing coverage. A BLASTn search was used to find two outgroup sequences; a *Miniopterus schreibersii* BHV (Bat BHV B7D8: JQ805139) and a Tupaiid BHV (NC_002794), and Clustal Omega (9) was then used to conduct sequence alignment. BEAST v2.5 was then used to produce phylogenies using an GTR+I+G substitution model. To estimate the divergence dates of DrBHV strains within Peru, the B7D8 bat BHV branch time was selected as a calibration point. Assuming that, due to co-divergence of BHVs with bat host species, the branch time for this divergence is the same as that for the branch time for the divergence of *Miniopterus* and *Desmodus*. The prior for this branch time was set at 45mya (10), with a normal distribution and a standard deviation of 5my, based approximately on the uncertainty of date of divergence between *Desmodus* and *Miniopterus*.

The divergence time for DrBHV within Peru was estimated to be 1.66mya (95% HPD 0.96-2.53mya). The most recent common ancestor (MRCA) to the second outgroup used in this tree of a Tupaiid herpesvirus 1 (NC_002794) was estimated to be 89.51mya (95% HPD 56.11-132.11mya), which reasonably reflects the predicted divergence time for Chiroptera and primates, of approximately 82mya (Upham et al., 2019). Since only 5/11 strains were observed as single infections and therefore possible to include in our molecular clock analysis, strain age could not be calculated for all strains.

In order to see if HCMV glycoprotein B genotypes showed a similar relationship between strain age and prevalence, several glycoprotein B sequences for each of the four main genotypes were collected from GenBank, as well as some for which the genotype had not been previously assigned (MK157451.1, KT987994.1, GU937742.2, KJ361951.1, KR992927.1, KT987995.1, FJ527563.1, KT987992.1, KT987993.1, FJ616285.1, KT987991.1, MK157427.1, KT987990.1, KT726950.2, KR992921.1, KR992910.1, MK157428.1, KT726951.2, MN274568.2, KT726955.2, KR992837.1, M60926.2, U88700.1). HCMV sequences were aligned along with a Panine HV2 (AF480884.1) sequence, for which a divergence time of 3.8mya was assigned. BEAST v2.5 was used as above to estimate divergence dates of HCMV glycoprotein B genotypes. These divergence dates revealed to HCMV gB4 to be the oldest strain, and data collated from several studies on HCMV prevalence (11–14) also revealed gB4 to be the least prevalent of the genotypes.

**Supporting references**

1. Bergner LM, Orton RJ, da Silva Filipe A, Shaw AE, Becker DJ, Tello C, et al. Using noninvasive metagenomics to characterize viral communities from wildlife. Molecular Ecology Resources. 2019 Jan;19(1):128–43.

2. Streicker DG, Winternitz JC, Satterfield DA, Condori-Condori RE, Broos A, Tello C, et al. Host–pathogen evolutionary signatures reveal dynamics and future invasions of vampire bat rabies. Proc Natl Acad Sci U S A. 2016 Sep 27;113(39):10926–31.

3. Peakall R, Smouse PE. GenAlEx 6.5: genetic analysis in Excel. Population genetic software for teaching and research—an update. Bioinformatics. 2012 Oct 1;28(19):2537–9.

4. Hijmans RJ. geosphere: Spherical Trigonometry. 2019.

5. Etten J van. R Package gdistance: Distances and Routes on Geographical Grids. Journal of Statistical Software. 2017 Feb 27;76(1):1–21.

6. Robert, J. Hijmans, Etten J van. raster: Geographic analysis and modeling with raster data. 2012; Available from: http://CRAN.R-project.org/package=raster

7. Yu G, Smith DK, Zhu H, Guan Y, Lam TT-Y. ggtree: an r package for visualization and annotation of phylogenetic trees with their covariates and other associated data. Methods in Ecology and Evolution. 2017;8(1):28–36.

8. Jari Oksanen, F. Guillaume Blanchet, Michael Friendly, Roeland Kindt, Pierre Legendre, Dan McGlinn, et al. vegan: Community Ecology Package. 2019; Available from: https://CRAN.R-project.org/package=vegan

9. Sievers F, Wilm A, Dineen D, Gibson TJ, Karplus K, Li W, et al. Fast, scalable generation of high-quality protein multiple sequence alignments using Clustal Omega. Mol Syst Biol. 2011 Oct 11;7:539.

10. Agnarsson I, Zambrana-Torrelio CM, Flores-Saldana NP, May-Collado LJ. A time-calibrated species-level phylogeny of bats (Chiroptera, Mammalia). PLoS Curr [Internet]. 2011 Feb 4 [cited 2020 May 13]; Available from: index.html%3Fp=387.html

11. Ciotti M, Cella E, Rittà M, Ciccozzi M, Cavallo R, Perno CF, et al. Cytomegalovirus Glycoprotein B Genotype Distribution in Italian Transplant Patients. INT. 2017;60(4):165–70.

12. Gonzalez-Sanchez HM, Alvarado-Hernandez DL, Guerra-Palomares S, Garcia-Sepulveda CA, Noyola DE. Cytomegalovirus glycoprotein B genotypes in Mexican children and women. Intervirology. 2015;58(2):115–21.

13. Sarkar A, Das D, Ansari S, Chatterjee RP, Mishra L, Basu B, et al. Genotypes of glycoprotein B gene among the Indian symptomatic neonates with congenital CMV infection. BMC Pediatr. 2019 Aug 22;19(1):291.

14. Taherkhani R, Farshadpour F, Makvandi M, Hamidifard M, Esmailizadeh M, Ahmadi B, et al. Determination of cytomegalovirus prevalence and glycoprotein B genotypes among ulcerative colitis patients in ahvaz, iran. Jundishapur J Microbiol. 2015 Feb;8(2):e17458.
