# Supplementary material for: Longitudinal deep sequencing informs vector selection and future deployment strategies for transmissible vaccines
Source: PLoS Biol. 2022 Apr 19;20(4):e3001580. doi: 10.1371/journal.pbio.3001580 (PMC9017877; doi:10.1371/journal.pbio.3001580)
Supplement: Attachment — Submitted filename: Reviewer response 4.docx [file pbio.3001580.s017.docx]

**Response to Reviewers**

Editor and reviewer comments are shown in black.

Our response to each comment is shown in blue.

**Editor comments**

You will see that the reviewers find your conclusions interesting and important for the field, however they also raise several concerns that would need to be addressed in order for us to consider the manuscript further for publication. Reviewer 1 mentions a general issue regarding the reliance on a small segment of glycoprotein, which will be important for you to address. Reviewer 2 would like you to clarify several points and to tone down some of the claims made in the abstract.

We have addressed the concerns of both reviewers; specifically, we have carried out additional analyses which make use of a far larger section of the genome, including both glycoprotein and non-glycoprotein sequences where possible. We have also clarified the pertinent sections of manuscript as requested, and toned down the relevant abstract claims. We thank both reviewers for their critiques, which have improved the manuscript.

**Reviewers' comments**

**Reviewer 1:**

In this paper, the authors extend their previous work investigating the suitability of Desmodus rotundus betaherpesvirus (DrBHV) as a vector for a transmissible vaccine targeting rabies in vampire bats. The primary advance of this work over previous results reported by this group (Griffiths et al. 2020) is the substantially increased scope of viral sequencing and the analysis of viral sequences collected from bats that were captured and later recaptured. The increased scope of viral sequencing allows the prevalence of individual strains to be established and the analysis of recaptured animals allows the temporal dynamics of viral infections and super infections to be studied. These are fundamental properties that must be understood in the development of any transmissible vaccine platform. The authors' analyses suggest that individual strains of DrBHV reach locally high prevalence and that superinfection is possible. These are both promising and important results. Although I am generally convinced by the authors' overall conclusions, I do have some questions and concerns about methods the authors use and the robustness of some of the specific claims made throughout. Below, I detail these questions and concerns.

The most obvious limitation of this work is the reliance on a 150 bp segment of a viral glycoprotein. This is a very small proportion of the viral genome that likely experiences strong (and potentially spatially and temporally variable) selection imposed by the host. Because of this, I was not convinced that: A) analyses of Fst for this region tell us much of anything about patterns of viral gene flow among regions, or that B) nucleotide diversity tells us much of anything about the age of a strain. In addition to violations of neutrality, the potential for recombination of this small region onto different viral genomic backgrounds also muddies the water with respect to these conclusions. I would like to see these issues addressed and their potential consequences for the authors' conclusions discussed. For instance, if nucleotide diversity has been shaped by repeated selective sweeps within strains, how would this influence the authors' conclusions?

We agree that basing our original analysis on a small portion of the glycoprotein B gene was not ideal. We previously focused on this segment (despite having sequenced a longer region) because the combination of short read sequencing and the unexpectedly high frequency of multi-strain infections within individual bats precluded construction of longer contigs which could unambiguously be joined together as part of the same virus strain. In the revised manuscript, however, we have adopted new analytical approaches that do not require all reads to be linked to the same genome. At least for some key analyses, this allowed us to take advantage of the full sequence data available. These changes are described in more detail below in response to the reviewer’s specific comments.

Figure 1b is a bit challenging to interpret quickly because colors indicate different things in the different panels. Explaining that the different panels in B are each showing one of the strains would help speed interpretation.

The caption for figure 1b has been updated to include a clearer explanation of the link between panels a and b: ‘Each map corresponds to a single strain, numbered S1 to S11 as in panel A.’ We also updated the strain labelling terminology to be consistent across panels of the figure.

Lines 144-147. I appreciate what the authors are trying to do here, but using pairwse Fst as an estimate of viral gene flow is tenuous at best because: 1) Fst depends on population size as well as gene flow and the authors estimates of strain specific prevalence suggest substantial differences in population size, 2) these results are gleaned from a 150bp fragment and so may not be representative of the genome as a whole, and 3) the glycoprotein is likely under selection that potentially varies spatially. I need to be convinced by the authors that this is a robust conclusion, because I currently am not.

To increase the amount of genetic data used in our analysis of viral gene flow, we took a new approach inspired by recent developments in the analysis of pooled next generation sequencing data, where data contain sequence reads from related, but non-identical genomes (i.e., “poolseq” (1)). In our data, ‘pooling’ consists of NGS reads which may represent different DrBHV strains within individual bats. Specifically, we used the R package ‘poolfstat’ to calculate pairwise Fst between geographical groups based on SNP read counts from the full 12kb sequenced region. To address the reviewer’s concern about selection, we carried out a similar analysis after removing the SNPs from the UL55 glycoprotein gene (full details can be found from pages 7 and 19; lines 181 and 501). The patterns identified previously are also observed in the new analyses, with the exception of Cajamarca now clustering more closely with Lima than with the other Northern departments of Amazonas, Huánuco, and Loreto. This change is consistent with an earlier population genetic analysis of vampire bats (2), strengthening our conclusion. Similar patterns are observed in analyses both with and without data from UL55 included, and continue to show far higher similarity to the host population structure than could be expected by chance. While we appreciate that F_ST_ may in theory be influenced by population size, we think that the evidence presented elsewhere in our manuscript is not consistent with this as the primary explanation for the patterns of apparent population structure observed. For example, multiple other entities (e.g., rabies virus and bat microsatellites) which may not necessarily have the same geographic pattern in population size, have the same population structure when analysed using different analyses (e.g., phylogenetic analyses or STRUCTURE). Thus, we are confident that the results of our analysis are most parsimoniously explained as a consequence of viral gene flow. Our new results from the poolseq approach are shown in a revised version of Figure 3 and described in pages 7-8, lines 186-202.

Lines 154-157. Does this really show host specificity? What about just general movement corridors used by multiple bat species? This seems consistent with host specificity but not demonstrative of host specificity.

We agree that this is not demonstrative of host specificity, and have changed the wording of this section accordingly.

Lines 162-174. "strain age (approximated as relative nucleotide diversity given that the nucleotide diversity of a strain would be expected to increase over time)." This seems extremely speculative and tenuous at best. Nucleotide diversity could be proportional to the age of a strain if evolution is purely neutral. But, even then, does the increase in nucleotide diversity within a strain not also depend on population size? Given the authors demonstration that strain prevalence (and presumably thus population size) varies wildly, is there actually any reason to expect that nucleotide diversity is indicative of age? This would not be true at equilibrium where we know the expected value of nucleotide diversity is equal to 4 * N * mu. Add onto this the very likely existence of selection and my confidence in this analysis becomes quite low. Here, too, I need to be convinced by the authors that this is a robust conclusion, because I currently am not.

We agree with the reviewer that nucleotide diversity is an imperfect proxy for strain age; however, we would expect that if nucleotide diversity predominately reflected current viral population sizes, then we should see a positive relationship between the nucleotide diversity of a strain and its estimated prevalence. Instead, we find the opposite where more genetically diverse strains have lower prevalence. While we acknowledge this is not evidence that variation in population size has no effect on nucleotide diversity, it also does not point to this obviously being a major problem for our analysis. In any case, the main issue here is in the interpretation of the relationship demonstrated, which itself is robust. As such we prefer to leave this analysis in, acknowledging its limitations. Specifically, we have added text to the discussion describing the caveats in interpreting the relationship between nucleotide diversity and prevalence, including the possible effects of selection and variation in viral population size (page 13, lines 332-344). To exclude the impact of selection specifically, we repeated the analysis using strains defined from a section of UL54, which should experience less selective pressure than UL55 (page 6, lines 159-165).

Lines 221-226. I do not follow the logic here. The two sentences seem directly contradictory. Specifically, how is this statement "bats with higher initial strain richness acquired fewer additional strains at later time points" consistent with this statement "with strain acquisition dependent on the number of locally available strains, rather than how many an individual is infected with." I must be missing something, but no matter how many times I read these they seem to directly contradict one another.

These lines have been re-written to clarify that it is the proportion of locally available strains the bat is not yet infected with – those available for acquisition, not the number of strains it is infected with at timepoint 1, that influences how many strains are gained (page 9, lines 230-240). The associated figure 4b has also been updated to clarify this point.

Lines 260-264. "A major concern with using naturally occurring, innocuous, and host-specific viruses as transmissible vaccine vectors has been that cross-immunity from natural circulation of wild-type ancestors could impede vaccine spread [3]." This is, of course, only part of the challenge. If a TV rapidly dumps its antigenic cargo then it must compete with a genetically identical strain that presumably has the advantage of no longer carrying the antigenic insert. This is obviously not something that can be addressed with a field study, but this should be clearly articulated here to make it clear we have much more to learn about the dynamics of superinfection with genetically identical strains.

We agree that competition between vaccine strains and their non-vaccine descendants is still a necessary component to investigate. We have expanded the discussion to include the problems that the loss of genetic insert could cause (pages 11-12, lines 296-300).

Lines 437-439. "Tree prior Coalescent: constant size, and strict clock were used, 1x107 generations run with a sampling frequency of 1000, and a burn-in of 10%." This is way outside my area of expertise, but what are the consequences of assuming a constant population size and clocklike evolution in a case where: 1) the authors argue lineages arise and expand (population size not constant) and 2) the GPC is a strong candidate for periodic selection and decidedly non-clocklike evolution?

The clock model and tree priors initially selected are the default ‘base models’ to which other models are compared. The analysis was run using both Constant and Bayesian Skyline tree priors, each combination for 1x10^6^ generations. Both models grouped the 11 strains in largely the same manner; however, we agree that a more flexible population model is more realistic, and now present the results of the Bayesian Skyline tree prior with strict clock model. Tip dates were not used for this analysis, due to the short time period over which they were collected compared to the timescale of the likely MRCA, and insufficient information was available to inform internal node priors. As such, branch lengths represent the number of substitutions per site and are not time scaled, so the choice of clock is less relevant for this analysis, and a strict clock was selected. The methods have been updated to reflect this (page 19, lines 490-500).

In summary, the core results of this work are robust and of broad interest. To the best of my knowledge, this is the most comprehensive study to date of the natural ecology and evolution of a betaherpesvirus that may prove to be a useful vector for a recombinant vector transmissible vaccine. The most important and novel results are the description of strain specific prevalence over space and the demonstration that superinfection appears to occur in individuals that have been studied over time. A weakness of this work, however, is that the authors over-interpret their results with respect to their more elaborate conclusions. I think the work would benefit from focusing on the robustly supported core results and eliminating the speculative conclusions about gene flow, strain dynamics over space and time, and boosting. This is not to say I believe those results are incorrect, just that the results presented here do not provide sufficiently robust support for these conclusions.

We appreciate the reviewer’s acknowledgement of the scale of the advance our work provides and the helpful comments which we believe have considerably strengthened the manuscript.

**Reviewer 2:**

Griffiths et al. have submitted the manuscript "Multi-strain dynamics and phylogeographic structure of a candidate viral vector supports the development of a transmissible vaccine for vampire bat rabies", which attempts to unravel the phylogeography, host-use and multi-strain dynamics of the Desmodus rotundus betaherpesvirus (DrBHV) in Peru. The authors overarching aim is to lay necessary groundwork for the potential suitability of DrBHV as a transmissible vaccine (a vaccine vectored by a naturally occurring, avirulent virus) for rabies.

In summary, the authors convincingly demonstrate that DrBHV is genetically heterogeneous, with at least 11 strains (of varying intra-strain heterogeneity) being found to circulate in bat populations across Peru. Strains were found to largely vary according to host population structure as well as elevation. In addition to a considerable prevalence of DrBHV, bats were investigated for multi-strain dynamics, revealing that multiple strains of the virus are often supported by hosts. A small number of bats were also examined chronologically, revealing that infections apparently become cleared or are (re)acquired over the course of a bat's lifetime.

However, the study does not fully evaluate whether the DrBHV system can be effectively exploited as a transmissible vaccine, as several important factors were not directly tested. The authors highlight some of these outstanding gaps in the discussion, which are very much appreciated, but mention of other issues in the discussion would be worthwhile. For example, is there experimental evidence showing that DrBHV is innocuous in bats? (This may be known, but reference to the relevant studies could be given). Can inserts be purged by selection? This could seem to be a potential issue with transmissible vaccines generally. If so, what steps could be taken to prevent insert loss in the DrBHV system? For readers unfamiliar with the topic, consideration of such issues would be useful. The abstract could also benefit from being toned down a bit, e.g. this sentence seems slightly overreaching "Our results catalyse the development of a transmissible, multi-year, self-boosting vaccine targeting the most important source of human and animal rabies in Latin America".

We thank the reviewer for these important points, which we agree are all outstanding questions and we plan to address each in future studies. In the revised version of the manuscript, we have added context and further details for all of the above areas in the discussion (pages 14;11 lines 379-382; 296-300). We have also toned down the abstract as requested.

Overall I support the publication of this interesting manuscript. In addition to the above contextual improvements, there are some points that the authors should consider to help clarify the ms.

Major points

The Mantel tests (or their description) seem fairly crucial to the study, but they are slightly difficult to interpret and/or appear at odds with the written text. This may be a misunderstanding on my part, but it could benefit from a bit of clarification. The first test compares "Fst values for DrBHV and host microsatellites, grouped by the Department from which sequences originated". The p-value is close to being significant, but this is not necessarily noteworthy. However, the second mantel test compares either Fst values of DrBHV or bat microsatellites with "least cost distance model outputs". It is not very clear which of the former it is because in the results it reads as though the DrBHV Fsts are used, whereas in the methods, the second test "compare the matrices of microsatellite Fst values grouped by bat colony, with the least cost distance model outputs for travel between colonies". This should be cleared up, and the second test is also highly significant

(P=1x10-4) so it is hard to reconcile it/interpret alongside the first mantel test.

The Fst analysis and Mantel tests have been re-analysed based on comments from Reviewer 1. The results of this section were therefore rewritten (pages 7-8, lines 179-202) with the different comparisons laid out more clearly, as well as the addition of Figure 3. We have also provided these results in greater detail the supplementary materials (S2 Table).

From the phylogeny in Fig. 1, it looks like there could be some "singleton" viral sequences that don't fit into the strain categories, e.g. the sequence at the very bottom of the phylogeny, sister to "S1" and a sequence between "S1" and "S2" that appears to be a singleton too. How were these treated, or is it just an issue with the figure graphic formatting?

The S1-related sequence was indeed an outlier. We suspect it could be the result of a recent recombination event which has yet to become fixed in the population, and was excluded from later strain analyses. This point is now discussed in the results (page 5, line 133). The S2-related sequences that the reviewer noted had been improperly formatted and this has been rectified in Figure 1.

On a similar theme, can strain divergence levels be somehow validated by comparing against existing full-genome sequences (if available, e.g. from single-strain infected bats)? I imagine this is probably not possible given current data availability, and the authors acknowledge the potential shortcoming of the strain ID in the discussion already, so this may be a moot point.

Unfortunately, non-chimeric genome sequences do not currently exist, so we are unable to validate these divergence levels at the genome level.

The precise (lat,long) sampling sites are also not given. This would be useful information for meta-analyses etc. Ditto regarding availability of qPCR data.

Latitude and longitude coordinates for each sampling site have been added to the supplementary materials (S1 Table), as well as which sample sites belong to which geographical groups. The qPCR data (Ct values) has been uploaded to an online repository which will be linked under data availability in the main text. ([https://figshare.com/articles/dataset/Betaherpesvirus_sequencing_and_qPCR_on_Peruvian_vampire_bat_saliva_samples_xlsx/15067884](about:blank))

Figure 2 analyses of strain age, range and prevalence are also very important to the study, and overall I find the correlations to be convincing. But what is the situation at a closer scale? From the methods (although this is also a little bit unclear), it reads as though there could be multiple sampling locations per group (within a 10km range). Was sampling carried out consistently in different "groups" and are similar patterns of strain prevalence/diversity observable on this finer sampling scale. E.g. wrt strain competition, are strain number and prevalence at individual sampling locations also uncorrelated? I mention it because transmission and competition dynamics might be more meaningful at that scale. Although I must also confess that my knowledge of vampire bat ecology is insufficient to argue this convincingly! Also I fully understand that it may not be possible to test this idea given the study's sampling design, but if it were somehow possible, it might be interesting to look at briefly. More detail about the sampling strategy (sites per group, lat/long locations, distances etc) wouldn't go amiss either.

We have added details about the sample sites and their grouping to the supplementary materials (S1 Table). We agree that strain competition at a finer scale may be important. We therefore repeated our analysis of strain number vs strain prevalence at the sample site level and show that there is still no significant interaction or evidence of strain competition. The results from this analysis are now discussed at page 6, lines 157-159.

Fig 2B also appears quite bimodal. What is happening there and can the model account for that?

The bimodal appearance of figure 2b may be the result of both actual strain distribution due to geographic barriers (mountains) that separate Peru into roughly North and South, and our sampling design. In the model, the GLMM residuals are normally distributed (Shapiro-Wilk test, p-value=0.661), indicating that results are robust to the bimodal explanatory variable. We also carried out a second check where we converted the convex hull areas for each strain into a binary measure of ‘high geographic coverage’ and ‘low geographic coverage’ and repeated the GLMM analysis. This showed a highly significant effect of this binary measure of viral geographic range on prevalence (Estimate=0.694, error=0.199, z-value=3.481, p-value= 5E-4).

I am somewhat agnostic about the inclusion of the dated HCMV phylogeny, as the age of the gB4 genotype is scarcely greater than some of the others, e.g. gB3. On the other hand I can also see the reasoning behind the analysis.

We accept the limitations of this analysis, but believe that it provides useful context nonetheless and have retained it in the supplementary materials.

Minor points

Line 106 typo "superinfection and the of"

Line 108 typo "vampire bats Peru"

Line 629. Oddly formatted reference 40

These typos have been corrected, and the reference reformatted.

Fig 1. A very minor point but the lineages and the geographic locations vary in (very broadly) opposite directions: i.e. uppermost lineages have a more southerly distribution and vice versa. Would be visually nice to reverse that somehow.

We have re-arranged the layout of this figure so that strain 1 is at the top in both panels A and B.

**References:**

1.	Hivert V, Leblois R, Petit EJ, Gautier M, Vitalis R. Measuring Genetic Differentiation from Pool-seq Data. Genetics. 2018 Sep;210(1):315–30.

2.	Streicker DG, Winternitz JC, Satterfield DA, Condori-Condori RE, Broos A, Tello C, et al. Host–pathogen evolutionary signatures reveal dynamics and future invasions of vampire bat rabies. Proc Natl Acad Sci U S A. 2016 Sep 27;113(39):10926–31.
