# Supplementary material for: Longitudinal deep sequencing informs vector selection and future deployment strategies for transmissible vaccines
Source: PLoS Biol. 2022 Apr 19;20(4):e3001580. doi: 10.1371/journal.pbio.3001580 (PMC9017877; doi:10.1371/journal.pbio.3001580)
Supplement: Attachment — Submitted filename: Reviewer comments_3.docx [file pbio.3001580.s018.docx]

**Response to Reviewers**

Reviewer comments are shown in black.

Our response to each comment is shown in blue.

**Reviewers' comments**

The authors have done a very comprehensive job of revising their manuscript and have directly addressed my previous concerns. Including the additional analyses based on pooled full-length sequences represents a significant investment by the authors in a thoughtful revision. We still disagree here and there on the strength of some specific conclusions, but this is now more a matter of opinion than anything else. There is just one last substantive issue that had not occurred to me during my original reading but which I can now not quite put to rest. This is the consequences of using affinity propagation to define strains as genetically similar clusters.  What is bothering me about this is that it seems it would place a cap on the possible amount of nucleotide diversity that could be found within a "strain" before it is split by the algorithm into a new strain. Thus, only genetically similar sequences could be widespread and prevalent without being split out into separate strains. Could this lead to spurious conclusions about relationships between prevalence, range, and genetic diversity? I don't know the answer here and could be way off base, but it might be worth thinking a bit about what the null relationship should be between nucleotide diversity and prevalence or range when strains are defined as genetically similar clusters. I suspect the correct null is not the absence of a relationship.

We appreciate the reviewer’s concern; however, the nature of our study required an operational definition of strains. In the absence of antigenic data, clustering by genetic information remains the only way to define viral strains, and those predicted by the clustering algorithm had strongly supported phylogenetic relationships. This approach also had the advantage of providing a repeatable operational definition, which we preferred over the use of arbitrary assignments. The reviewer is correct that strain definitions ultimately depend on the observed data and taxonomy may be updated as new viruses are discovered, but this issue is not unique to our study.

To investigate the null relationships between prevalence, strain range and strain nucleotide diversity, we conducted a randomization test, in which tree tips were randomly reassigned location meta-data, and geographic range, nucleotide diversity, number of strains per group, and group specific strain prevalence recalculated. This was repeated 1000 times, and the statistical results (Beta, standard error, z-value, and p-value) recorded for each randomization (S2 Data). The observed test statistics from the real data used in our initial analysis were found to fall outside of the 95% distribution of the null model. This randomization test show that, under our initial strain definition, the observed patterns are very unlikely in the absence of real relationships between prevalence and strain nucleotide diversity and geographic range.

We have amended Figure 2 to include the average null relationship from our randomization test (lines 155-157 and 171-179). We have also further discussed why our strain definition was necessary, and acknowledged that geographic range of strains is likely to be sensitive to the way in which strains are defined and the data available (lines 376-385).
